# Supplementary material for: Demonstration of an always-on exchange-only spin qubit
Source: Nat Commun. 2026 Apr 3;17:4794. doi: 10.1038/s41467-026-70943-w (PMC13219449; doi:10.1038/s41467-026-70943-w)
Supplement: Supplementary file 1 — Supplementary Information [file 41467_2026_70943_MOESM1_ESM.pdf]

Supplementary Information for: Demonstration of an always-on exchange-only spin qubit

Joseph D. Broz,[1,*] Jesse C. Hoke,[1] Edwin Acuna,[1] and Jason R. Petta[1,2,3,†]

[1] *HRL Laboratories, LLC, 3011 Malibu Canyon Road, Malibu, California 90265, USA*
[2] *Department of Physics and Astronomy, University of California – Los Angeles, Los Angeles, California 90095, USA*
[3] *Center for Quantum Science and Engineering, University of California – Los Angeles, Los Angeles, California 90095, USA*

I. DEVICE TUNE-UP

1-J control with each of the exchange axes is illustrated in Fig. S1. We plot the probability to return to state $|0\rangle$, $P_{|0\rangle}$, as a function of virtual plunger gate detunings (e.g. $\tilde{V}_{\mathrm{P}_1} - \tilde{V}_{\mathrm{P}_2}$) and virtual exchange gate voltages (e.g. $\tilde{V}_{\mathrm{X}_{1,2}}$). The $P_{|0\rangle}$ oscillation frequency increases with $\tilde{V}_{\mathrm{X}_{i,j}}$, as expected

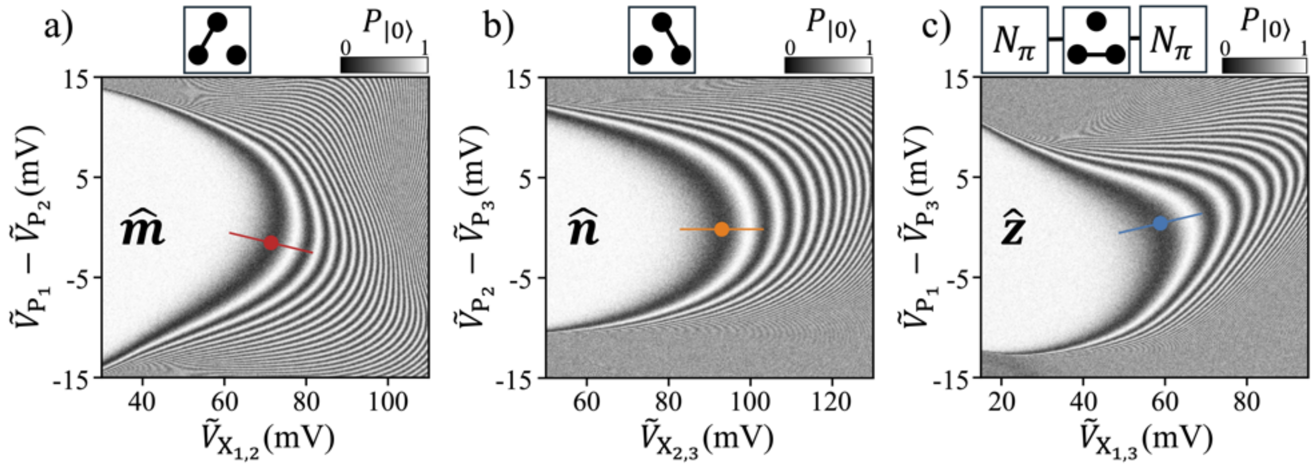

FIG. S1. **1-*J* exchange fingerprints**. (a) – (c) 1-J fingerprint plots for the $\hat{m}$, $\hat{n}$, and $\hat{z}$ axes. Colored circles indicate the approximate locations of the 1-J sweet spots where the exchange coupling is first-order insensitive to chemical potential fluctuations. The 1-J exchange rotations used throughout this work were calibrated along the symmetric axes of operations (colored lines), along which this condition is maintained. Pre/post π-rotations about the $\hat{n}$-axis are applied in (c).

II. 2-J GATE CALIBRATION

The Hamiltonian for an exchange-only (EO) qubit can be written as:

$$\hat{H} = -\frac{1}{2}[\sqrt{3}J_-\hat{\sigma}_x + (J_{1,3} - J_+)\hat{\sigma}_z], \tag{1}$$

where $\hat{\sigma}_i$ are the standard Pauli operators, and $J_+ = (J_{1,2} + J_{2,3})/2$ and $J_- = (J_{1,2} - J_{2,3})/2$. An exchange pulse of duration τ generates a rotation by an angle of θ about an axis $\mathbf{r} = (r_x, r_y, r_z) = (\cos(\varphi), 0, \sin(\varphi))$ in the xz-plane of the Bloch sphere, as described by the unitary operator:

$$\hat{R}_\varphi(\theta) \approx \cos(\theta/2) - i\sin(\theta/2)\mathbf{r}\cdot\hat{\boldsymbol{\sigma}}, \tag{2}$$

* jdbroz@hrl.com
† jpetta1@hrl.com

where $\hat{\boldsymbol{\sigma}} = (\hat{\sigma_x}, \hat{\sigma_y}, \hat{\sigma_z})$, $\theta = \int_0^\tau \Omega(t)dt$, and:

$$\Omega = \sqrt{3J_-^2 + (J_{1,3} - J_+)^2}, \tag{3}$$

$$\cos(\varphi) = \sqrt{3}J_-/\Omega, \tag{4}$$

$$\sin(\varphi) = (J_{1,3} - J_+)/\Omega. \tag{5}$$

The values of θ and φ are determined by the strength, $J_{i,j}$, of the pairwise exchange interactions activated during the pulse (Eqs. 3 – 5), which, in turn, depend on the amplitude of the voltage pulses applied to the gate electrodes. For 1-J exchange, φ is restricted to the discrete set $\{0, 2\pi/3, 4\pi/3\}$, corresponding to the $\{\hat{z}, \hat{n}, \hat{m}\}$ axes shown in Fig. 1(b) of the main text. In contrast, for 2-J exchange pulses, φ can be tuned to an arbitrary value.

To calibrate a 2-J rotation $\hat{R}_{\varphi^*}(\theta^*)$, corresponding to target angles φ^* and θ^*, we employ the composite sequence $\hat{U}(N)$, where:

$$\hat{U}(N) = \hat{U}_{\text{ax}}^N \hat{U}_{\text{ang}}^N, \tag{6}$$

$$\hat{U}_{\text{ax}} = \hat{R}_\varphi^{2q}(\theta), \tag{7}$$

$$\hat{U}_{\text{ang}} = (\hat{R}_\eta(\chi)\hat{R}_\varphi^q(\theta))^2, \tag{8}$$

and the integer q is chosen such that $q\theta^* = s\pi$ for some odd integer s. The operator $\hat{R}_\eta(\chi)$ is a pre-calibrated rotation with parameters $\eta \approx \varphi^* + \pi/2$ and $\chi \approx \pi$, and is assumed to be implemented using a single 1- or 2-J exchange pulse [1]. The sequence $\hat{U}(N)$ is designed to reduce to the identity when $\hat{R}_\varphi(\theta)$ is perfectly calibrated, i.e.,

$$\hat{U}(N; \varphi^*, \theta^*) = \hat{\mathbb{1}}. \tag{9}$$

Furthermore, $\hat{U}_{\text{ax}}$ is designed to amplify deviations from Eq. 9 due to errors ϵ_φ in the axis angle, $\varphi = \varphi^* + \epsilon_\varphi$, by a factor that scales with the repetition number N. While $\hat{U}_{\text{ang}}$ similarly amplifies errors ϵ_θ in the rotation angle θ. In the terminology of gate set tomography, $\hat{U}_{\text{ax}}$ and $\hat{U}_{\text{ang}}$ are called germs and N the germ power [2]. By twirling $\hat{U}$ over the set of 1-J single-qubit Clifford gates $\mathcal{C}$, we can directly measure the fidelity, $F(\hat{U}, \hat{\mathbb{1}})$, of $\hat{U}$ relative to the identity [3, 4]:

$$F(\hat{U}, \hat{\mathbb{1}}) = \frac{1}{|\mathcal{C}|} \sum_{i=1}^{|\mathcal{C}|} |\langle 0|\hat{C}_i^\dagger \hat{U} \hat{C}_i|0\rangle|^2. \tag{10}$$

In practice, each term in the summation is obtained by initializing the qubit in $|0\rangle$, applying the sequence $\hat{C}_i^\dagger \hat{U} \hat{C}_i$, and then measuring the probability that the qubit remains in $|0\rangle$ [5]. By measuring F across a range of the exchange gate voltages used to implement $\hat{R}_\varphi(\theta)$ we generate two-dimensional plots similar to those shown in Fig. 4 of the main text. These data exhibit a series of peaks, but only the central peak corresponds to the optimal calibration point where $\varphi = \varphi^*$ and $\theta = \theta^*$. To track this peak, we perform successive sweeps with increasing values of $N = 1, 2, 4, 8, 16, \ldots$, continuing until reductions in the signal-to-noise ratio prevents further scaling. Practically, charge noise limits us from setting N much larger, but we note that already with $N = 24$ we are able to estimate the peak bias values at a precision comparable to the limitations of our control hardware ($\sim 7\ \mu$V). At each stage, we select the peak nearest to the value identified in the previous step [6]. This iterative approach is similar to the approach used in robust phase estimation [7], and the entire protocol can be viewed as a two-dimensional generalization of that technique. While not strictly necessary, we find that twirling $\hat{U}$ enhances the contrast of the interference peaks and suppresses the effects of time-correlated noise.

To accurately determine the location of the central calibration peak using this protocol, we fit the measured data to the analytical expression (e.g. red contours in Fig. 4 of the main text):

$$\begin{aligned} F(\hat{U}, \hat{\mathbb{1}}) = 1 - \frac{2}{3}\Bigg\{ \Bigg[\cos(N\theta) + \Big((r_x k_z - r_z k_x)^2 + k_y^2 \Big) \sin^2(N\theta/2) \Bigg] S_{2N}[\sin^2(\Phi/2)] \\ + \frac{1}{2}(r_x k_x + r_z k_z) \sin(N\theta) U_{4N-1}[\cos(\Phi/2)] \sin(\Phi/2) + \sin^2(N\theta/2) \Bigg\}. \end{aligned} \tag{11}$$

Here, S_M are spread polynomials of order M [8], which can be expressed in terms of the Chebyshev polynomials of the first kind as T_M as $S_M(x) = [1 - T_M(1 - 2x)]/2$, and U_M are Chebyshev polynomials of the second kind. The parameters Φ and $\mathbf{k} = (k_x, k_y, k_z)$ characterize the net rotation resulting from the composition $\hat{R}_\chi(\eta)\hat{R}_\varphi(\theta)$:

$$\hat{R}_\chi(\eta)\hat{R}_\varphi(\theta) = \cos(\Phi/2) - i\sin(\Phi/2)\mathbf{k} \cdot \hat{\boldsymbol{\sigma}}, \tag{12}$$

with explicit expressions:

$$\cos(\Phi/2) = \cos(\chi/2)\cos(\theta/2) - \sin(\chi/2)\sin(\theta/2)\cos(\varphi-\eta), \tag{13}$$

$$\sin^2(\Phi/2) = \cos(\chi/2)\sin(\theta/2) + \sin(\chi/2)\cos(\theta/2)\cos^2(\varphi-\eta) + \sin^2(\chi/2)\sin^2(\varphi-\eta), \tag{14}$$

$$k_x = \frac{\cos(\varphi)\cos(\chi/2)\sin(\theta/2) + \sin(\chi/2)\cos(\theta/2)\cos(\eta)}{\sin(\Phi/2)}, \tag{15}$$

$$k_y = -\frac{\sin(\theta/2)\sin(\chi/2)\sin(\varphi-\eta)}{\sin(\Phi/2)}, \text{ and} \tag{16}$$

$$k_z = \frac{\sin(\chi/2)\cos(\theta/2)\sin(\eta) + \sin(\varphi)\cos(\chi/2)\sin(\theta/2)}{\sin(\Phi/2)}. \tag{17}$$

When fitting the data, we relate the rotation parameters φ and θ to the exchange energies of the 2-J pulse $\hat{R}_\varphi(\theta)$ using using Eqs. 3 – 5. The exchange energies themselves are modeled as having an independent exponential dependence on the exchange gate voltages, described by $J_{i,j} = A\exp[B\tilde{V}_{X_{i,j}} + C]$, where A, B, and C are fit parameters. We find that the validity of this last assumption improves as the range over which the exchange gate voltages are swept decreases. In practice, we only fit the final sweep ($N = 24$ for the data presented in the main text) to Eq. 11. In the preceeding sweeps, we use a heuristic algorithm to locate the peak: first applying a Gaussian filter to the data, then thresholding at 80% of the maximum value, and finally computing the centroid, which we identify as the peak. In Figs. S2–S4, we use Eq. 11 to illustrate some important features of the calibration procedure, specifically the scaling with N, and the effects of calibration errors on η and χ.

We conclude with two remarks on the generality of this procedure. First, while we assumed in this analysis that the pre-calibrated rotation $\hat{R}_\eta(\chi)$ was constructed from a single 1- or 2-J exchange pulse, it may be realized as a composite pulse sequence. In that case, an additional error term must be considered in the analysis to account for possible deviations of the rotation axis from the xz-plane. However, the advantage of using such composite sequences is that they allow the use of pre-calibrated 1-J gates to tune 2-J rotations about an arbitrary axis in the xz-plane. Thus, this procedure directly extends to the more general case. Second, as in robust phase estimation, our calibration procedure is limited to the calibration of rotation angles θ that are rational multiples of π [7]. However, it should be possible to extend this procedure to arbitrary θ by calibrating a sequence of rotations about, say, $\pi/10, \pi/9, \pi/8, \ldots, \pi$, about some axis and then using fits of the data near each of these peaks to perform nonlinear interpolation as is done in the 1-J case [9].

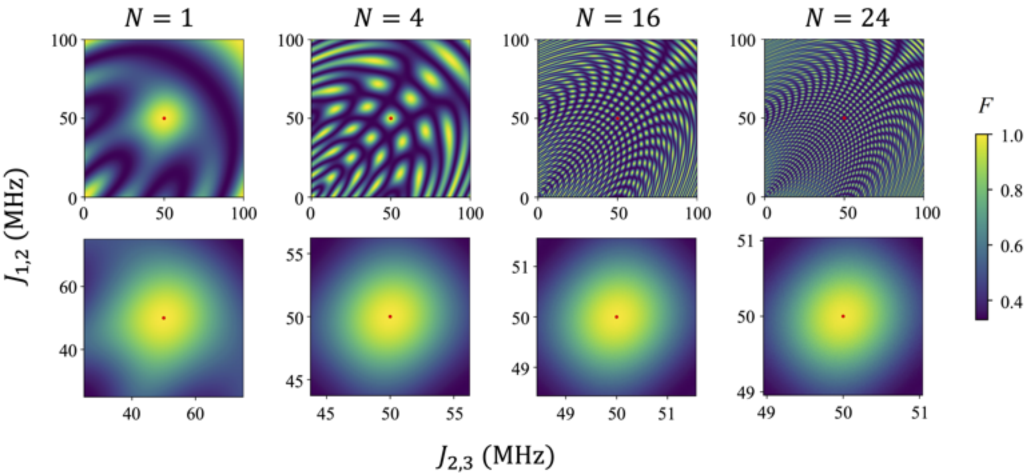

FIG. S2. **Scaling of the central calibration peak with N.** We consider the calibration of a 2-J rotation $\hat{R}_{\varphi^*}(\theta^*) = \hat{R}_{-\pi/2}(\pi)$, which corresponds to a π-pulse about the $-\hat{z}$ axis. In this case, the rotation parameters of the pre-calibrated pulse $\hat{R}_\eta(\chi)$ are: $\eta = \pi$, and $\chi = \pi$. The plots show the functional dependence of F (Eq. 11) on the exchange energies $J_{1,2}$ and $J_{2,3}$ for several different values of N. The relationship between φ and θ on $J_{1,2}$ and $J_{2,3}$ are given by Eqs. 3, 4. The bottom row are zoom-ins near the central peaks of the top row. In each plot, the red marker indicates the location of the optimally calibrated 2-J pulse, $\hat{R}_{\varphi^*}(\theta^*)$, which occurs when $J_{1,2} = J_{2,3} = 50$ MHz. Evidently, the size of the central peak reduces linearly with increasing N.

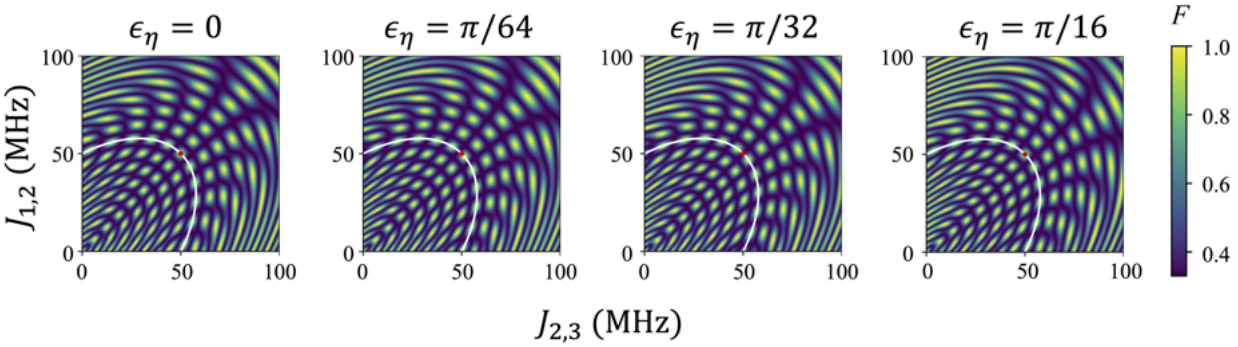

FIG. S3. **Effect of errors in η.** As in Fig. S2, we evaluate F (Eq. 11) for a 2-J π-rotation about the $-\hat{z}$ axis. Here, we fix $N = 8$ and consider the effects of errors on the rotation axis of the pre-calibrated pulse $\hat{R}_\eta(\chi)$: $\eta = \varphi + \pi/2 + \epsilon_\eta$. The white curve is a contour of constant $\theta = \theta^* = \pi$. The red marker indicates the location of the optimally calibrated 2-J pulse, $\hat{R}_{\varphi^*}(\theta^*)$, which occurs when $J_{1,2} = J_{2,3} = 50$ MHz. Errors, $\epsilon_\eta \neq 0$, cause the central peak to shift along the contour of constant θ. Along this contour, the interference peaks have a periodicity of $\pi/2N = \pi/16$. Effectively, an error ϵ_η causes an equal but opposite error in the calibration of φ.

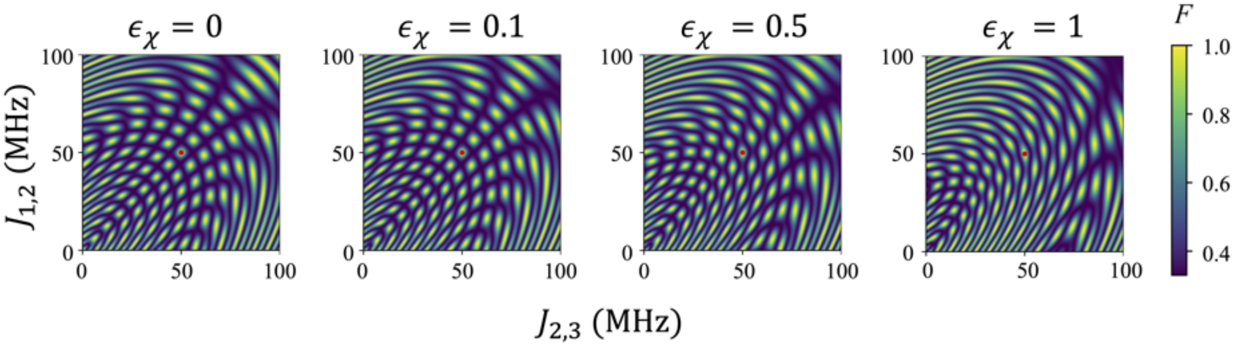

FIG. S4. **Effect of errors in χ.** As in Fig. S2, we evaluate F (Eq. 11) for a 2-J π-rotation about the $-\hat{z}$ axis. Here, we fix $N = 8$ and consider the effects of errors on the rotation angle of the pre-calibrated pulse $\hat{R}_\eta(\chi)$: $\chi = \pi + \epsilon_\chi$. The red marker indicates the location of the optimally calibrated 2-J pulse, $\hat{R}_{\varphi^*}(\theta^*)$, which occurs when $J_{1,2} = J_{2,3} = 50$ MHz. The central peak is relatively robust to small errors in $\epsilon_\chi \neq 0$. Moreover, these errors only cause a distortion of the shape of the central peak and not a shift of its mean location. In practice, we leave χ as a free parameter when fitting to data.

III. INTERLEAVED BLIND RANDOMIZED BENCHMARKING (BRB)

Following the calibration procedure, we tune nine distinct 2-J exchange pulses corresponding to rotations of $\pi/2$, π, and $3\pi/2$ about each of the axes: $\hat{x}$, $-\hat{x}$, and $-\hat{z}$. The locations of these pulses in bias space are indicated by the colored markers in Fig. 2 of the main text. To evaluate the performance of each pulse, we perform interleaved BRB, which involves interleaving a calibrated 2-J rotation into BRB sequences constructed from 1-J exchange pulses [9]. The data are shown in Fig. S5 and the extracted gate errors are summarized in Table S1. Interestingly, the analysis of the interleaved BRB results indicate the average leakage error per 2-J pulse is typically negative, implying that the total leakage error of the sequence is reduced when 2-J pulses are interleaved, despite the increased number of total pulses. This effect is not fully understood, but may arise from a refocusing mechanism similar to that observed when applying dynamical decoupling sequences in triple quantum dot spin qubits [10].

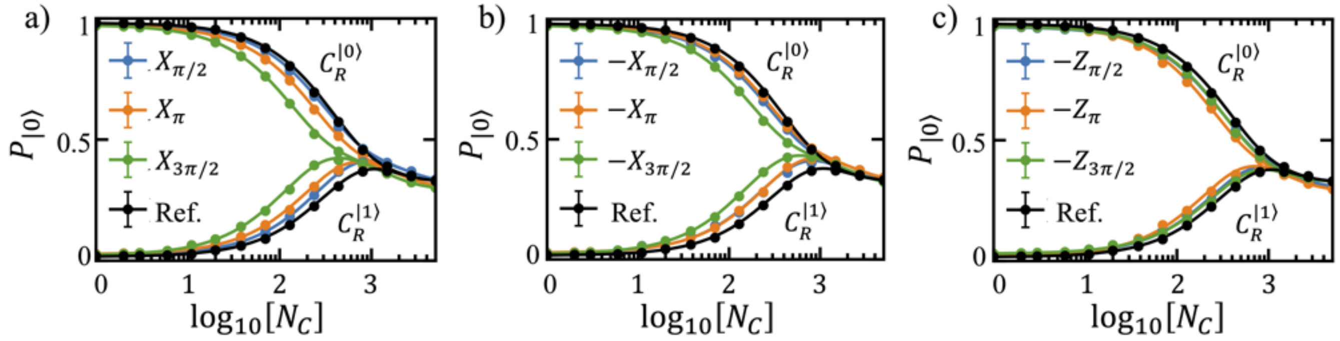

FIG. S5. **Interleaved blind randomized benchmarking**. (a) – (c) Results of interleaved BRB for 2-J $\pi/2$, π, and $3\pi/2$ rotations about the $\hat{x}$, $-\hat{x}$, and $-\hat{z}$ axes. The 2-J rotations are interleaved between random sequences of 1-J Clifford gates of depth N_{C1}. The reference corresponds to standard BRB using sequences of depth N_{C1} composed of only 1-J Clifford gates. Standard BRB analysis is performed on the resulting data. The interleaved gate errors and interleaved leakage errors are estimated by subtracting the reference sequence errors from the fitted errors of the interleaved sequences. The results of these calculations are summarized in Table S1. Error bars indicate the standard error of the mean probability from 250 sequence repetitions.

Axis	Angle	Total Error (10^{-3})	Leakage Error (10^{-3})
$\hat{x}$	$\pi/2$	0.240	-0.095
	π	0.794	-0.018
	$3\pi/2$	2.48	0.044
$-\hat{x}$	$\pi/2$	0.500	-0.547
	π	0.387	-0.102
	$3\pi/2$	1.55	-0.069
$-\hat{z}$	$\pi/2$	0.326	-0.005
	π	1.30	-0.210
	$3\pi/2$	0.306	-0.078

TABLE S1. Summary of 2-J total gate error and leakage errors extracted from interleaved blind randomized benchmarking.

[1] For the gates calibrated in the main text, we used a 1-J π pulse about the $\hat{z}$-axis to calibrate 2-J rotations about the $\hat{x}$-axis and used a 2-J π pulse about the $\hat{x}$-axis to calibrate 2-J rotations about the $-\hat{z}$-axis.

[2] E. Nielsen, J. K. Gamble, K. Rudinger, T. Scholten, K. Young, and R. Blume-Kohout, Gate Set Tomography, Quantum **5**, 557 (2021).

[3] E. Magesan, J. M. Gambetta, and J. Emerson, Scalable and robust randomized benchmarking of quantum processes, Phys. Rev. Lett. **106**, 180504 (2011).

[4] E. Magesan, R. Blume-Kohout, and J. Emerson, Gate fidelity fluctuations and quantum process invariants, Phys. Rev. A **84**, 012309 (2011).

[5] Sequences corresponding to different $\hat{C}_i$ are randomly ordered and typically we repeat each measurement for ∼10 shots to estimate the probability.

[6] We initialize the procedure using a coarse estimate of the peak location based on "fingerpinch" sweeps similar to those shown in Fig. 2 of the main text.

[7] S. Kimmel, G. H. Low, and T. J. Yoder, Robust calibration of a universal single-qubit gate set via robust phase estimation, Phys. Rev. A **92**, 062315 (2015).

[8] N. J. Wildberger, *Divine Proportions: Rational Trigonometry to Universal Geometry* (Wild Egg Books, Sydney, Australia, 2005).

[9] R. W. Andrews, C. Jones, M. D. Reed, A. M. Jones, S. D. Ha, M. P. Jura, J. Kerckhoff, M. Levendorf, S. Meenehan, S. T. Merkel, A. Smith, B. Sun, A. J. Weinstein, M. T. Rakher, T. D. Ladd, and M. G. Borselli, Quantifying error and leakage in an encoded Si/SiGe triple-dot qubit, Nat. Nanotechnol. **14**, 747 (2019).

[10] B. Sun, T. Brecht, B. H. Fong, M. Akmal, J. Z. Blumoff, T. A. Cain, F. W. Carter, D. H. Finestone, M. N. Fireman, W. Ha, A. T. Hatke, R. M. Hickey, C. A. C. Jackson, I. Jenkins, A. M. Jones, A. Pan, D. R. Ward, A. J. Weinstein, S. J. Whiteley, P. Williams, M. G. Borselli, M. T. Rakher, and T. D. Ladd, Full-permutation dynamical decoupling in triple-quantum-dot spin qubits, PRX Quantum **5**, 020356 (2024).
